# Quantifying fish otolith mineralogy for trace-element chemistry studies

**DOI:** 10.1038/s41598-022-06721-7

**Published:** 2022-02-17

**Authors:** R. Seth Wood, Bryan C. Chakoumakos, Allison M. Fortner, Kat Gillies-Rector, Matthias D. Frontzek, Ilia N. Ivanov, Linda C. Kah, Brian Kennedy, Brenda M. Pracheil

**Affiliations:** 1 Environmental Sciences Division, Oak Ridge National Laboratory, Oak Ridge, TN 37830 USA; 2 Department of Earth and Planetary Sciences, University of Tennessee, Knoxville, TN 37916 USA; 3 Neutron Scattering Division, Oak Ridge National Laboratory, Oak Ridge, TN 37830 USA; 4 Department of Fish and Wildlife Sciences, University of Idaho, Moscow, ID 83844 USA; 5 Center for Nanophase Materials Science, Oak Ridge National Laboratory, Oak Ridge, TN 37830 USA; 6 Present Address: Department of Earth and Planetary Sciences, Washington University in St. Louis, St. Louis, MO 63130 USA

**Keywords:** Biological techniques, Structural biology, Animal migration, Conservation biology, Stable isotope analysis

## Abstract

Otoliths are frequently used to infer environmental conditions or fish life history events based on trace-element concentrations. However, otoliths can be comprised of any one or combination of the three most common polymorphs of calcium carbonate—aragonite, calcite, and vaterite—which can affect the ecological interpretation of otolith trace-element results. Previous studies have reported heterogeneous calcium carbonate compositions between left and right otoliths but did not provide quantitative assessments of polymorph abundances. In this study, neutron diffraction and Raman spectroscopy were used to identify and quantify mineralogical compositions of Chinook salmon *Oncorhynchus tshawytscha* otolith pairs. We found mineralogical compositions frequently differed between otoliths in a pair and accurate calcium carbonate polymorph identification was rarely possible by visual inspection alone. The prevalence of multiple polymorphs in otoliths is not well-understood, and future research should focus on identifying otolith compositions and investigate how variations in mineralogy affect trace-element incorporation and potentially bias environmental interpretations.

## Introduction

The calcified structures of aquatic organisms such as otoliths (calcium carbonate CaCO_3_ ear stones), scales, shells, and fin rays are natural recorders of developmental history and environmental conditions. These structures incorporate trace-elements from ambient water into their CaCO_3_ matrix and permit temporally explicit environmental and ecological inferences about organismal migrations, habitat use, environmental contamination, and water temperature based on changes in trace-element concentrations among annular growth rings^1–3^. For example, large increases in otolith strontium (Sr) in diadromous fishes can be indicative of movement from freshwater to saltwater of the ocean and can be temporally contextualized based on their position relative to annular growth rings (e.g.^4–6^).

Otoliths are the most common calcified structure from fish used to reconstruct environmental conditions, and increased availability of analytical instruments has expanded the study of otolith microchemistry considerably over the past decade^2,7^. Although a variety of calcified structures from fish (e.g., scales, fin rays, cleithra, vertebrae, operculum) incorporate trace-elements from water and contain discernable growth structures, otoliths are considered the most robust recorder of environmental histories because they are metabolically inert and once accreted, are generally thought not to be resorbed by fish^8^. Nevertheless, a growing body of work suggests otolith mineralogy may need to be considered prior to assessment as they may contain any one or combination of the three most common CaCO_3_ polymorphs—aragonite (most common), vaterite, and calcite^9,10^. Although more extensive surveys of otolith compositions are needed, up to 48% of wild-produced fish populations and 80–100% of captive-reared fish populations may contain otoliths with non-aragonite polymorphs, excluding species like sturgeon and paddlefish which regularly have non-aragonite otoliths^9,10^. Each of these CaCO_3_ polymorphs has a unique crystallographic structure and trace-element affinities, meaning that under identical environmental conditions, trace-element concentrations may vary due to mineralogy alone^11,12^. In magnitude, polymorph-dependent changes in trace-element concentrations can mimic the changes associated with ecological events such as migrations. For instance, aragonite and vaterite portions of European eel (*Anguilla anguilla*) otoliths have differing strontium (Sr) concentrations that are like those associated with migrations between marine and freshwater systems^13^. The frequency of otoliths comprised of multiple CaCO_3_ polymorphs is poorly described at both a species and individual level, but otoliths with multiple polymorphs do not appear restricted to specific taxa or clades^10^ and have been linked to environmental factors^14–17^. Where multiple polymorphs have been reported for a fish species, it appears that any combination of CaCO_3_ polymorphs is possible within an individual otolith, otolith pair, and among populations (e.g.^13,14,18–21^).

Previous studies have shown that trace-element concentrations are similar between left and right otoliths of a pair and are therefore unlikely to affect environmental interpretations^22,23^, but it is unclear how varying proportions of CaCO_3_ polymorphs in otoliths of a pair may affect trace-element concentrations and, subsequently, affect environmental interpretations. This study has two primary objectives: (1) use neutron diffraction to identify and quantify CaCO_3_ polymorph abundances in otolith pairs from wild Chinook salmon *Oncorhynchus tshawytscha* which visually classified as non-aragonite (i.e., no otoliths in this study were identified as “normal” and comprised entirely of aragonite); (2) summarize a range of analytical techniques that can be used to qualitatively and quantitatively identify calcium carbonate polymorphs and aid otolith trace-element chemistry studies.

## Results

**Figure 1:**
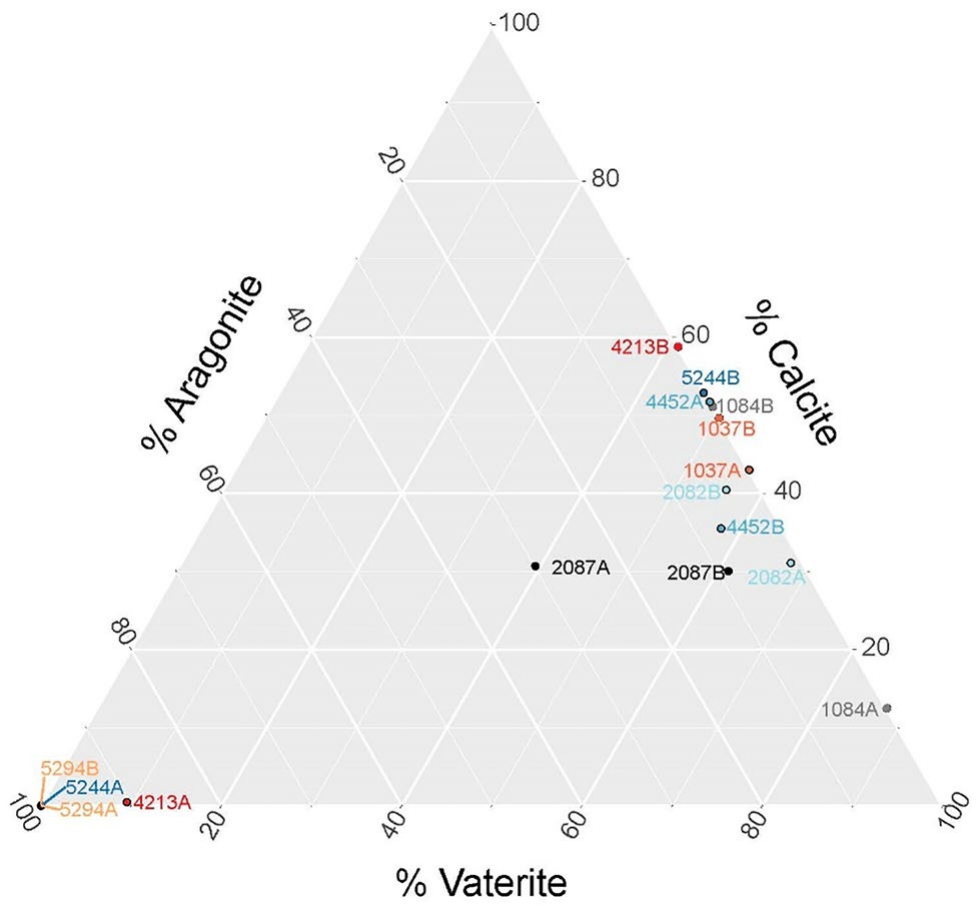
Ternary plot showing percentages of aragonite, calcite, and vaterite in each otolith pair. Otolith pairs can be identified by the text having the same color and same ID number with an A or B.

Wide-Angle Neutron Diffraction (WAND) was used to determine the presence and weight percent of CaCO_3_ polymorphs of bulk otoliths. The Goodness-of-fit values (*Χ*^2^ values) of WAND Rietveld refinements were > 10 for the otolith pair in fish 2082 and otolith 'B' for fish 2087 and 5244, which was beyond our arbitrary threshold for a “good” fit (*Χ*^2^ < 10 were deemed ‘good’). Among the remainder of analyzed otoliths, the percent weight for aragonite, calcite, and vaterite ranged from 1–100%, < 1–53%, and 9–88%, respectively. Among otoliths, all three of the most common CaCO_3_ polymorphs were identified, and 75% of the fish had at least one calcite-bearing otolith (Table 1; Fig. 1). Seven otoliths contained all three polymorphs, four of which came from two otolith pairs (although fish 2082 and 2087 had at least one otolith with poor goodness-of-fit for Rietveld refinements). In our sample set, calcite and vaterite always co-occurred in otoliths with more than one polymorph. Aragonite was the least common polymorph in the sample population but was also the only phase that comprised 100% of any individual otolith; only three otoliths were exclusively composed of aragonite, two of which were part of a pair. Two pairs of otoliths and one individual otolith contained no aragonite.

**Table 1:** Chi-squared goodness-of-fit information from Rietveld refinement of neutron diffraction data (*Χ*^2^ < 10 is a good fit) and results of aragonite, calcite, and vaterite percent fractions from each otolith ± SD for a pair of otoliths.

Fish ID	*X*^2^_A_	*X*^2^_B_	% Aragonite A	% Aragonite B	% Calcite A	% Calcite B	% Vaterite A	% Vaterite B
1037	4	4.57	0	0	43 ± 2	50 ± 2	57 ± 2	50 ± 2
1084	4	7.08	0	0	12 ± 1	51 ± 2	88 ± 3	49 ± 2
2082	11.6	52.8	1 ± 2	4 ± 1	31 ± 2	40 ± 2	68 ± 4	56 ± 2
2087	2.97	16.6	30 ± 1	9 ± 2	31 ± 1	30 ± 2	39 ± 2	61 ± 4
4213	2.22	5.72	90 ± 2	0	< 1 ± 1	59 ± 3	9 ± 1	41 ± 3
4452	3.83	1.97	0	7 ± 2	52 ± 2	36 ± 3	48 ± 2	58 ± 4
5244	3.03	18.7	100 ± 2	< 1 ± 2	0	53 ± 4	0	47 ± 4
5294	2.39	2.07	100 ± 2	100 ± 2	0	0	0	0

‘A’ and ‘B’ are used to denote parts of a pair because information on otolith positioning (i.e., left versus right) was not available.

**Figure 2:**
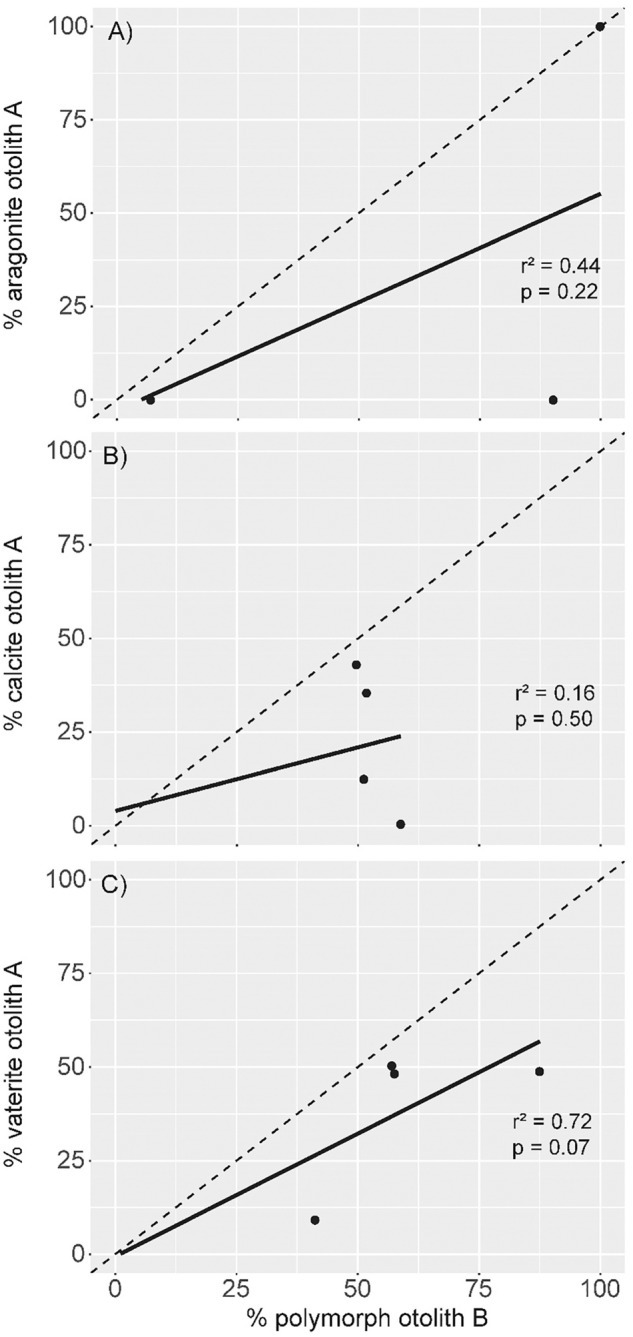
Scatterplot depicting the by-weight percentage of each polymorph in a pair of otoliths for aragonite (top panel), calcite (middle panel), and vaterite (bottom panel). The solid line represents the least-squares regression line for the samples and the dashed line represents the 1:1 line that would depict the regression line for the samples if ‘A’ and ‘B’ otoliths had identical polymorph composition. Each panel also contains r2 and associated p-values values for regression between ‘A’ and ‘B’ otoliths for each polymorph.

Although there is a visual difference between slopes of regression lines constructed between the composition of otoliths in a pair and a 1:1 line, the slopes of these lines were not significantly different for any of the CaCO_3_ polymorphs (Fig. 2). Among polymorphs, fractional vaterite abundance was most strongly correlated between otoliths in a pair, although it was not significant. Paired t-tests show significant differences between composition of calcite (t = 2.73, p = 0.03, 7 d.f.) and vaterite (t = 3.52, p = 0.01, 7 d.f.) for otoliths in a pair. There was not a significant difference in the percentage of aragonite between otoliths in a pair.

## Discussion

Like some previous studies, WAND data suggest that one or both otoliths in a pair can contain aragonite, vaterite and/or calcite, but this is the first study to quantify polymorph abundances of bulk otoliths in a pair^10^. Rietveld refinement—a technique for fitting structural models to XRD and neutron diffraction data—is essential for quantifying the polymorphic composition of bulk otoliths. While previous studies have reported polymorph composition(s) for left and right otoliths based on optical microscopy, Raman spectroscopy, and scanning electron microscope (SEM) analyses^24^ these techniques are superficial, and polymorph identification via optical microscopy and SEM is qualitative and have not been validated^10^. Given the complex, 3-dimensional distribution of polymorphs in some otoliths, surficial assessments, like Raman, may be inadequate for estimating phase abundances^25–27^. Other studies have employed X-ray diffraction (XRD) to identify CaCO_3_ phases in otoliths, but none have collected XRD data from otolith pairs and used Rietveld refinement to quantify the relative abundances of CaCO_3_ polymorph^10,23,25–27^. Although XRD and Raman are quantitative techniques, inferences about bulk otolith mineralogy are qualitative and speculative unless Rietveld refinements of the data were completed, or, in the case of Raman, an entire otolith was assessed—which is impractical given the minute excitation volume of a Raman laser (typically a few microns in depth, see “Methods”).

While previous studies have given valuable insight into the mineralogic variability of otoliths, optical and spectroscopic analytical techniques can only characterize the exposed, uppermost portion of an otolith and are often qualitative. For example, Gauldie^14^ reported no differences between the amount of vaterite replacement between left and right otoliths of Chinook salmon based on visual inspection and qualitatively described degrees of vaterite replacements on a scale of 1—4. Similarly, Tomas and Geffen^28^ used micro-Raman and image analysis to identify polymorphs and calculate the surficial abundance of vaterite in otoliths, respectively; XRD was also used in to confirm the presence of CaCO_3_ polymorphs, but Rietveld refinement was not used to quantify polymorph abundances for entire otoliths.

To highlight the potential frequency of mineralogically ‘aberrant’ otoliths and utility of bulk quantitative phase analyses, otoliths from 14 species of fish have been assessed with either XRD or neutron diffraction to quantify polymorph phase abundances (Rietveld refinement); among these studies, calcite was identified in more than 25% of species (lake sturgeon *Acipenser fulvescens*^9^, shovelnose sturgeon *Scaphirhynchus platyrhynchus*^10^, Chinook salmon (this study), goldeye *Hiodon alosoides*^25^). Excluding the present study, none of the assessed otoliths were visually identified as aberrant prior to assessment, and calcite was previously unreported for all four of these species. In contrast, when superficial or qualitative analytical techniques (including XRD without Rietveld refinement) have been used, calcite was only identified in otoliths from 11 of 94 fish species^10^.

**Figure 3:**
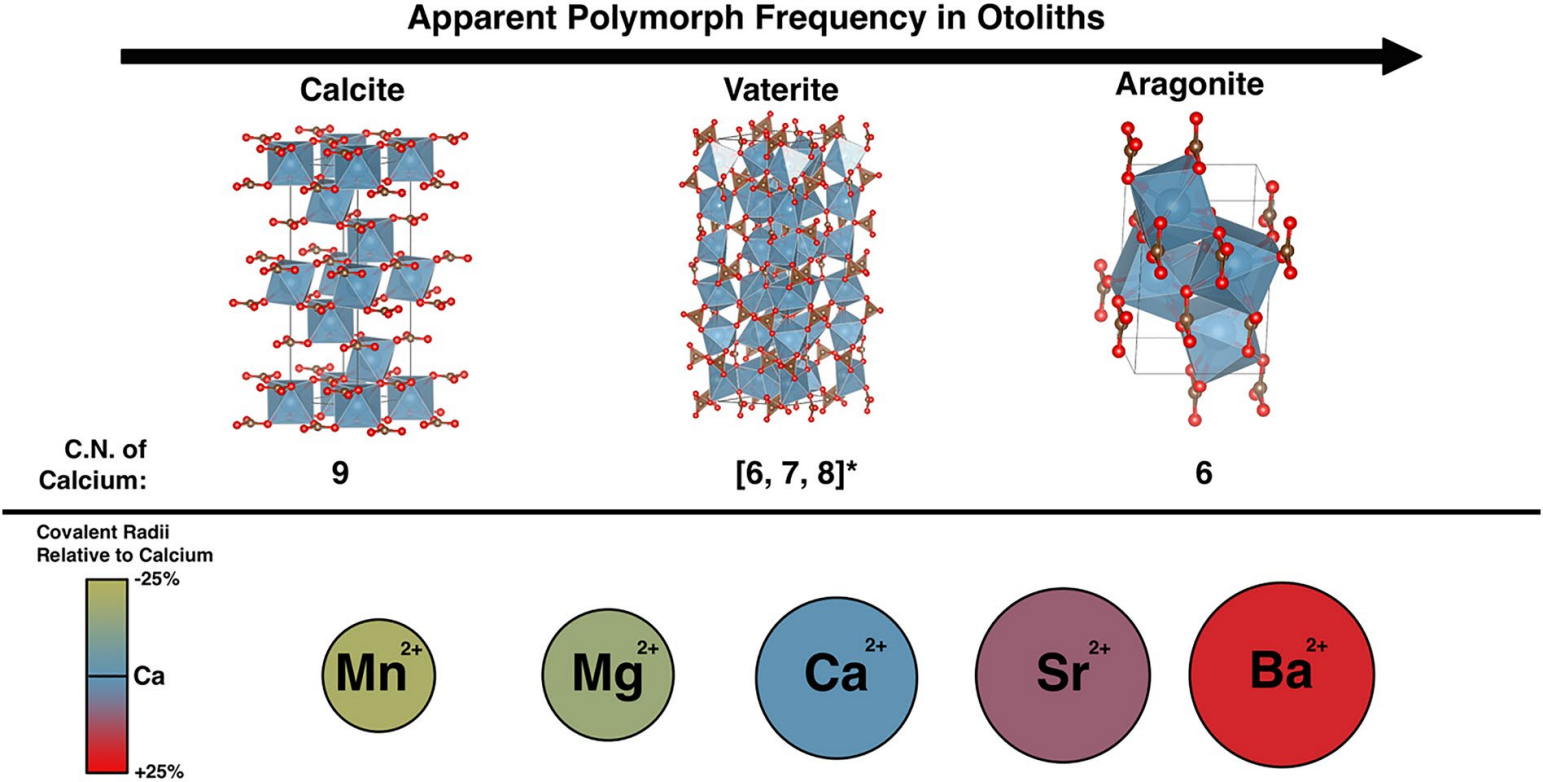
(Top) Crystal structures of calcite, vaterite, and aragonite unit cells^39^ in order of increasing apparent frequency in fish otoliths. Blue, brown, and red colors polyhedra correspond to calcium, carbon, and oxygen atoms, respectivley. Cordination number (C.N.) of calcium are listed below each polymoprh; astrisk (*) beside the caclicum coordination numbers for vaterite correspond to the P6_5_22 space group model^26^. Empirical covalent bond radii of cations (bottom) are relative to the covalent bond radius of calcium and shown for illustrative purposes^40^. Substitution of calcium with cations of larger or smaller radii will distort the crystalographic lattice of CaCO_3_ polymoprhs.

As non-aragonite otoliths are identified in more species, the need to understand how trace elements are partitioned between calcite and aragonite or calcite and vaterite becomes increasingly critical for interpreting microchemistry data. Figure 3 illustrates differences in the crystal structure and coordination numbers of Ca in CaCO_3_ polymorphs will affect how readily cations substitute for Ca^9,13,29^. Moreover, controlled precipitation experiments indicate sulfate (SO_4_^2−^) is more likely to substitute for carbonate (CO_3_^2−^) in calcite than aragonite, even though high sulfate concentrations can promote vaterite precipitation^30^. Controlled precipitation experiments also suggest trace element concentrations vary with calcite growth rate^31^, and within the optimal temperature range of a species, otolith accretion rates can double every 10 °C^32,33^. If trace element incorporation scales non-linearly as a function of growth rate among CaCO_3_ polymorphs, trace element measurements may need to be normalized to both host mineralogy and growth rate for accurate environmental inferences. At this time, it is unclear if differences in trace-element concentrations between calcite and aragonite or calcite and vaterite portions of an otolith are meaningful, but Sr concentrations in aragonite and vaterite could bias environmental inferences^13^.

Overall, the cause(s) and potential effects of calcite precipitation in otoliths are poorly resolved. To date, the most direct evidence for trace element partitioning between calcite, aragonite, and vaterite is from Pracheil et al.^10^ who used simultaneous X-ray fluorescence and XRD to assess Chinook salmon otoliths; the assessed otolith was comprised of all three CaCO_3_ polymorphs, and Sr concentrations were measurably higher in calcite regions than in vaterite but seemingly lower than aragonite (note that aragonite and calcite regions may have precipitated asynchronously and therefore changes in Sr concentration may reflect temporal changes in environmental conditions and/or mineralogy). Some experimental studies indicate fish growth rates^15^, ambient water temperature^16,34^, dissolved carbon dioxide^35^, protein expression^36^, and ontogeny^17^ affect CaCO_3_ polymorph expression in otoliths, but, in general, mechanistic associations between polymorph presence and environmental and genetic factors are not known. Unfortunately, due to the relatively small sample size of this study, we were unable to explore relationships between extrinsic factors, such as those shown in Table 2, and polymorph expression.

**Table 2:** Collection and life history information on Chinook salmon from which otoliths analyzed in this study were collected including date of fish collection, back calculated (from otolith) year of hatch, fish fork length at time of collection, age at time of collection, sex, and number of years the fish lived in freshwater or ocean habitats as determined by otolith strontium concentrations.

Fish ID	Collection date	Hatch date	Fork length (cm)	Age	Sex	Yrs freshwater	Yrs ocean
1037	10/27/2015	2013	75	2	F	0	2
1084	10/27/2015	2012	92	3	F	0	3
2082	11/03/2015	2012	87	3	F	0	3
2087	11/03/2015	2011	89	4	F	0	4
4213	11/17/2015	2011	87	4	F	0	4
4452	2012	2009	73	2	M	NA	NA
5244	11/09/2015	2012	83	3	M	1	2
5294	11/17/2015	2011	84	4	M	0	4

All fish collected for this study were presumed to be of wild origin due to absence of hatchery-implanted coded wire tags (but see “Fish collection” in “Methods”).

**Figure 4:**
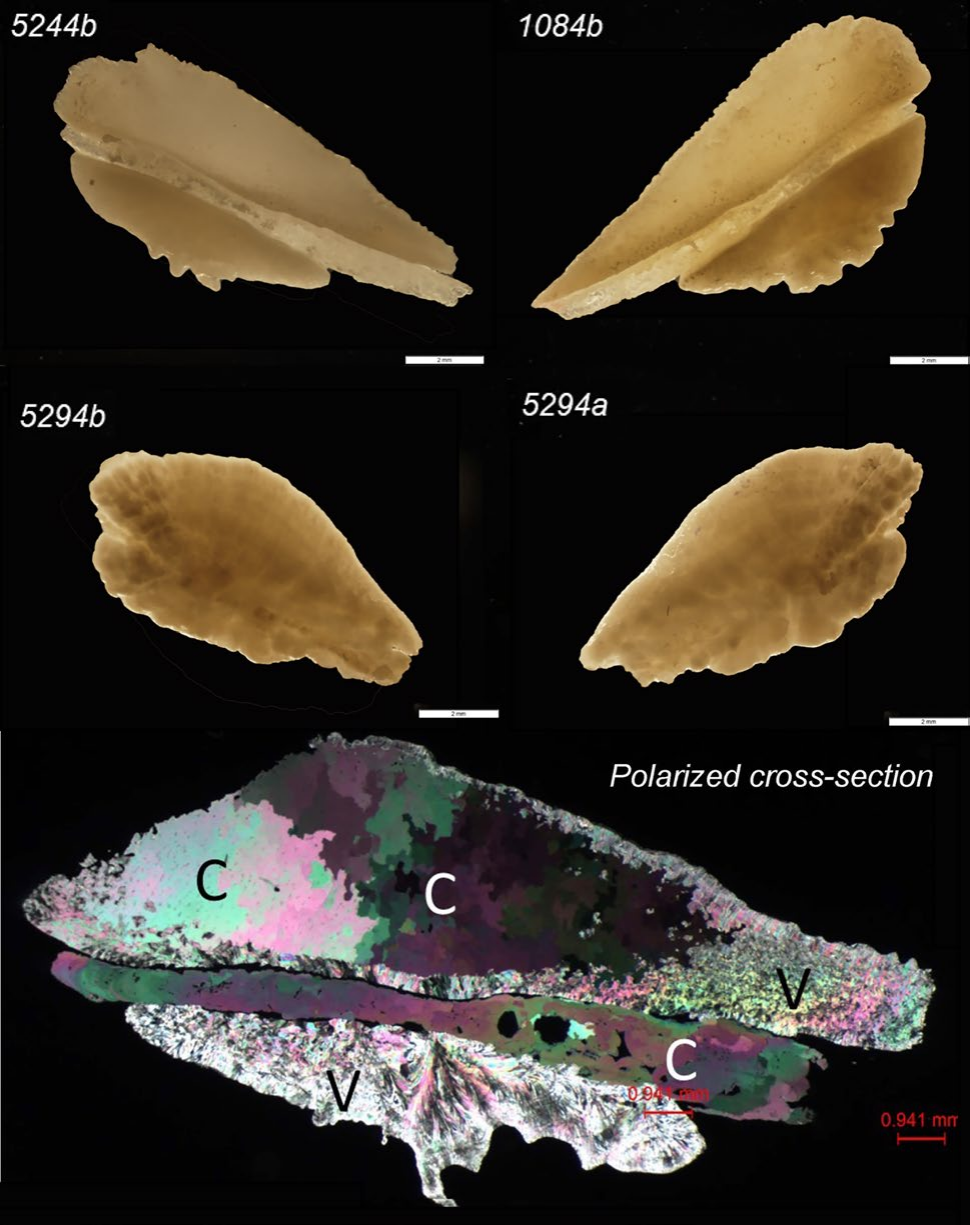
Proximal (top) and distal (bottom) micrographs of Chinook salmon otoliths comprised of calcite and aragonite from fish 1084 and 5244 and two all aragonite otolith from fish 5294. The polarized cross-section panel shows a prepared (30 μm thick) thin section of 5244-b Chinook salmon otolith under cross-polarized light microscopy showing areas of calcite (C) and vaterite (V). The calcite is readily identified by its uniaxial (−) optic sign and large grain-sizes relative to vaterite.

More focused investigations on otolith compositions are needed. The literature suggests transmitted light microscopy may be sufficient for identifying vaterite in some otoliths^10^. However, otoliths comprised of multiple CaCO_3_ polymorphs, like those reported in this study, can have a complex 3-dimensional distribution of polymorphs that cross-cut growth rings (Fig. 4) and transmitted light microscopy may be inadequate for identifying vaterite and/or calcite inclusions. We find standard petrographic thin sections (30 μm thick) of otoliths can aid the identification of calcite inclusions when assessed with cross-polarized light (Fig. 4). When otolith microchemistry will be used to inform conservation or management decisions, the presence and location of vaterite and/or calcite within an otolith may be critical for interpreting trace element concentrations^10^. Considering the broad lack of understanding about the occurrence of CaCO_3_ polymorphs in otoliths, the factors influencing polymorph expression, and the potential affects mineralogy may have on trace element concentrations, we conservatively recommend that all otoliths from individual fish, species, and populations be screened for vaterite and calcite prior to trace-element analyses.

**Figure 5:**
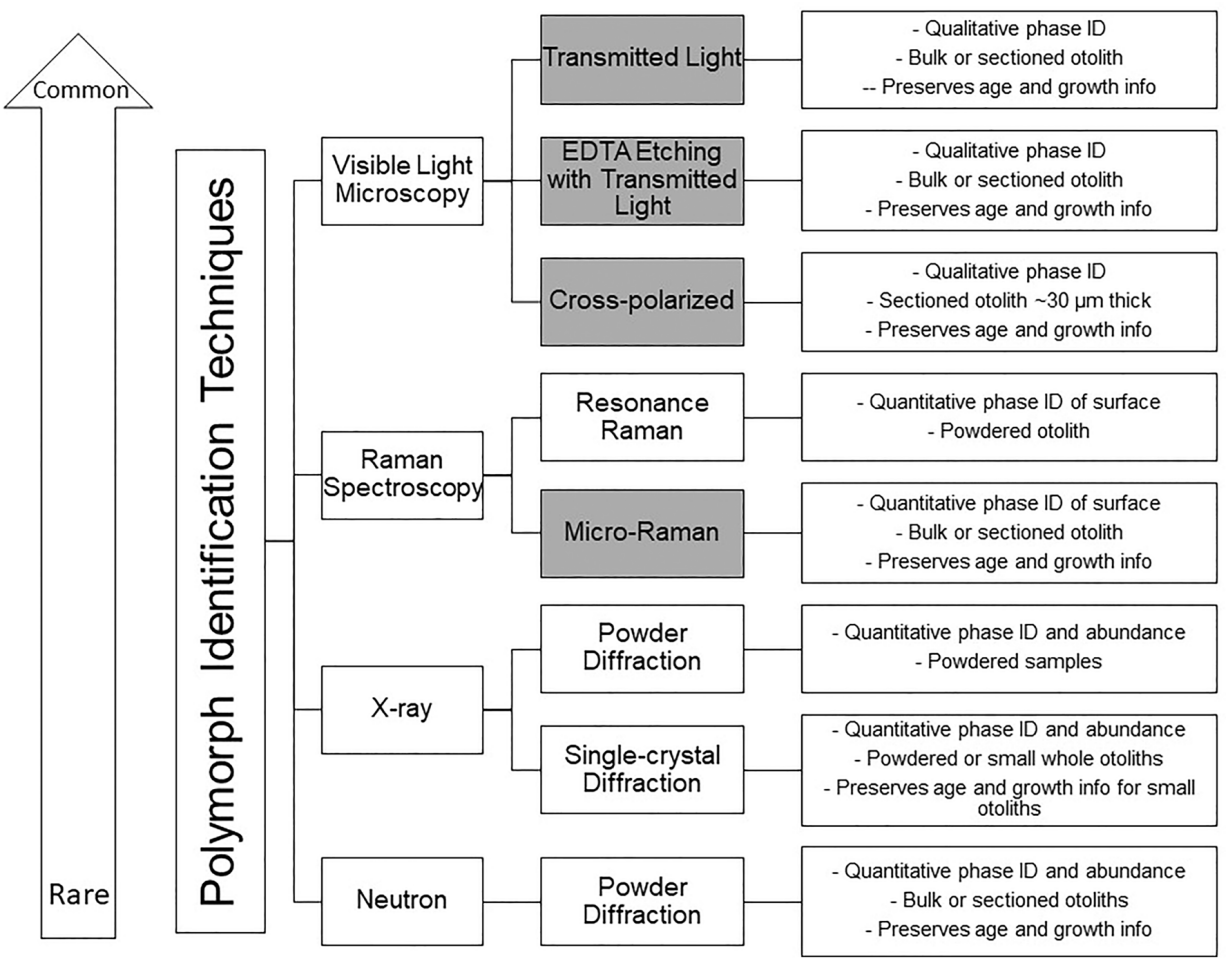
Analytical techniques that have been previously used in the literature for otolith polymorph identification with a short description of what information each technique can provide in the far-right boxes. Techniques are oriented in descending order of commonality. For example, visible light microscopy is available in most laboratories while neutrons are only available at a few locations worldwide. Gray boxes indicate techniques that provide spatially resolved polymorph identification where as hollow boxes indicate that techniques are not spatially resolved. However, please note discussion section for caveats associated with techniques presented in this figure.

We recognize that some methods for polymorph determination may be unfamiliar to fisheries biologists, so we have created Fig. 5 to assist investigators in screening otoliths. The choice of method may depend on what is most easily accessible. In this study polymorph abundances were quantified from neutron diffraction data, but micro-Raman can be used for superficial polymorph identification and is non-destructive (see “Methods”). X-ray diffraction and Rietveld refinement can be used to quantify the relative abundance of CaCO_3_ polymorphs in whole otoliths, but this technique requires an otolith be crushed and fully homogenized (thereby obscuring annual growth rings and limiting temporal inferences) or sufficiently small that the X-ray beam encompass the entire otolith and be diffracted from the interior. These instruments and petrographic microscopes are available at many universities in geology, chemistry, biochemistry, and materials science departments. These departments may also host LA-ICP-MS, TOF–SIMS, and other analytical instruments that can be used for spatially resolved microscale trace-element measurements. The identification and quantification of polymorphs in otoliths can be time-consuming and costly, and for some studies transmitted light microscopy may suffice. However, until the prevalence of non-aragonite otoliths is better understood and/or how trace-element are partitioned between calcite and aragonite or calcite and vaterite is known, we encourage researchers to perform more rigorous mineralogic assessments to further the study of otolith microchemistry (Fig. 5).

## Methods

### Fish collection

Otoliths used in this study were archival, and no live fish were handled by the authors or expressly for the purpose of this study. However, we present fish collection methods used by hatchery employees that collected otoliths used in this study as part of normal hatchery operations for environmental and ecological context of the sources of the otoliths used in this study. Sagittal otoliths from Chinook salmon (Fig. 4) were collected during the 2012 and 2015 hatchery spawning operations at Lyon's Ferry Hatchery, Washington, USA. Each year the hatchery captures a minor subset of the returning fall Chinook salmon population at Lower Granite Dam on the Snake River for propagation and subsequent restocking. Captured fish were assumed to be natural in origin, but a subset of hatchery-raised Chinook salmon was intentionally released without hatchery-identifying markings to supplement the naturally spawning population. Otoliths were collected from Chinook salmon that had a mean fork length of 84 cm, a mean age of 3 (range, age 2–4), and five of eight fish were female (Table 2). One fish was collected in 2012, and the remaining seven were collected in 2015. Fish spent 2–4 years in the ocean and 0–1 years in freshwater. Unfortunately, we do not have information on the prevalence of “abnormal” otoliths from the related study for which these otoliths were collected.

Otoliths recovered from captured fish were cataloged and organized by spawn date each harvest-year. Otoliths with semi-transparent growth rings identified in visual inspections were classified as vateritic and separated from non-vaterite otoliths. A subset of these vaterite otoliths were analyzed and no otoliths identified as “normal” (i.e., apparently made of aragonite only based on visual inspection) were included in this study. Information on whether otoliths came from the left or right sides was not retained, but otoliths from a single fish were stored together. Each otolith in a pair was arbitrarily labeled A or B for assessment purposes and do not necessarily correspond to the left or right side of a fish.

We focused on otoliths identified as vateritic during visual inspection because little is known about the quantitative abundance of vaterite in otoliths or the mineralogical variability of otoliths in a pair when vaterite is present. While vaterite otoliths may be more common within and among species than previously thought, they still appear to be relatively rare overall^9^. Neutron diffraction acquisition times averaged 2–3-h per otolith (although recent upgrades have reduced acquisition time to 45-min to 1-h) and limited the number of otolith pairs assessed during the allotted instrument time. Thus, only otoliths pairs suspected to contain vaterite were assessed in order to quantify vaterite abundances in individual otoliths and mineralogic heterogeneities within otolith pairs. Additionally, visual identification is commonly used as the only screen for vaterite in otoliths prior to a microchemistry study. Since neutron diffraction is deeply penetrating, it is uniquely well suited for quantifying bulk otolith mineralogy; data from neutron diffraction can therefore be used to evaluate the efficacy of visual mineral identification in otoliths.

### Neutron diffraction

**Figure 6:**
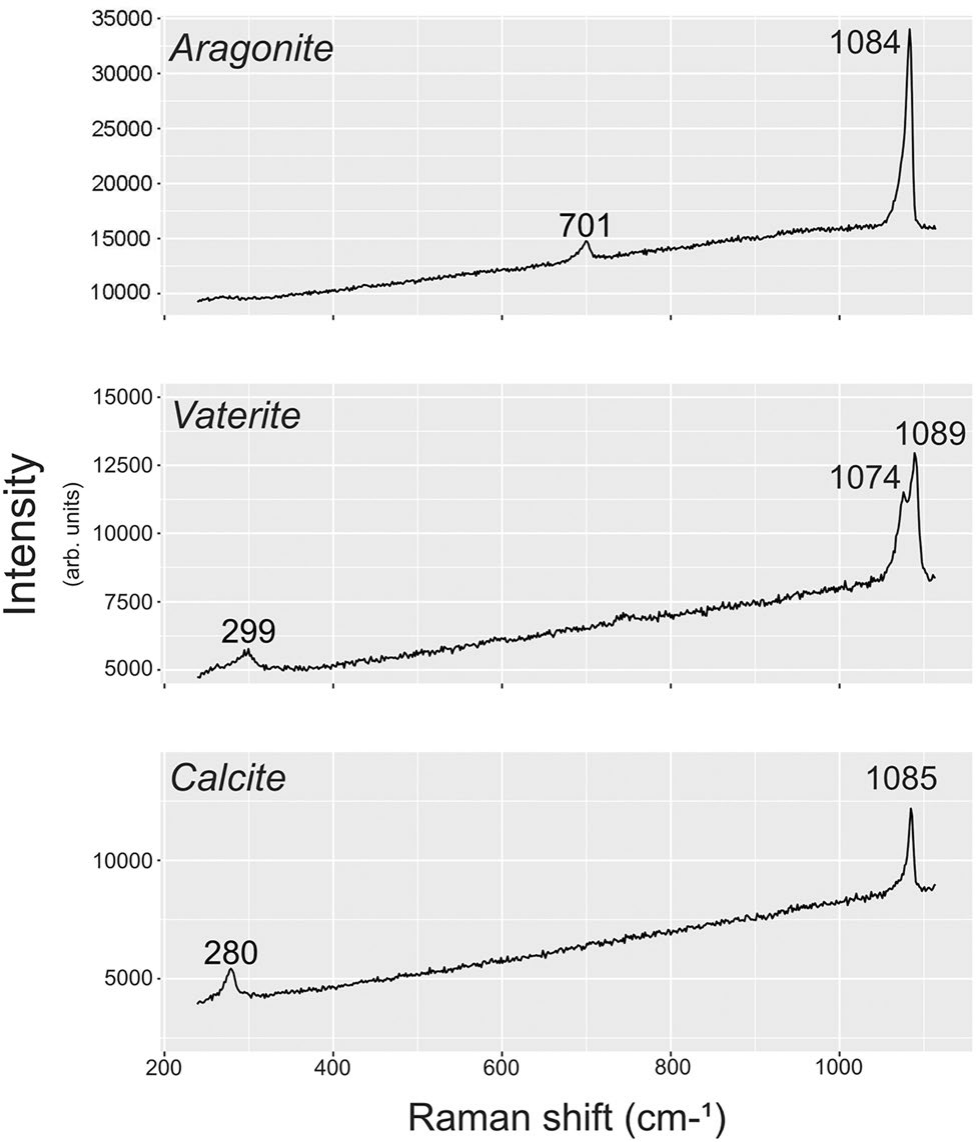
Typical Raman spectra depicting signatures of aragonite (top), vaterite (middle), and calcite (bottom).

The Wide-Angle Neutron Diffractometer (WAND) instrument at Oak Ridge National Laboratory’s High-Flux Isotope Reactor (HFIR) was used to differentiate and quantify CaCO_3_ polymorphs in each otolith of a pair (Fig. 6). WAND bombards the target otolith with neutrons of a known wavelength, and the angle and intensity of diffracted neutrons are recorded by an arced detector bank. Unlike X-ray diffraction or Raman spectroscopy, neutrons can penetrate an entire otolith, enabling bulk, quantitative mineralogic assessment. In contrast to micro-Raman spectroscopy, neutron diffraction does not provide information regarding the spatial distribution of polymorphs.

Otoliths were adhered to a short strand of monofilament fluorocarbon or an aluminum rod coated with neutron absorbing material using cyanoacrylate glue for WAND assessment. Once adhered, the aluminum road/monofilament was attached to a motorized gimbal that slowly rotated the otolith in front of the neutron beam. The motorized gimbal is used to mitigate potential crystal orientation affects—where crystals preferentially grow in a single direction. Each otolith was scanned four times with the WAND for a cumulative scan time greater than one hour. Two of the four scans measured diffraction angles between 5 and 130 2θ and the other scans measured diffraction angles between 35 and 160 2θ. Otolith masses were generally 50 to 100 mg, and in total, eight pairs of Chinook salmon otoliths (N = 16 otoliths) were assessed with the WAND.

### Neutron diffraction data analysis

Neutron diffraction data sets (i.e., scans recording the same range of diffraction angles on a single otolith) were combined, normalized to incident beam monitor counts, and binned in 0.1-degree intervals. Data for each otolith were exported in a format suitable for FullProf Suite 3.0^37^, which was used to refine phase abundances by the Rietveld method. The Automatic Background point tool in WinPLOTR^38^ was used to identify and fit background scattering. The Automatic Background tool consistently produced a background curve comprised of 15–20 points.

Crystallographic data for calcite, aragonite, and vaterite were taken from ICSD 73446, ICSD 15194, and Chakoumakos et al.^23^, respectively, and entered in EdPCR 2.0—a graphical editor in the FullProf Suite. During refinements, atomic positions and atomic displacement parameters were held fixed. The linearly interpolated background points were refined along with peak shapes, lattice parameters, scale factors, phase fractions, and detector bank zero offset.

Initially, otolith in a pair were presumed mineralogically homogenous, and identical refinement parameters were used for each otolith in a pair, excluding background points. This methodology frequently produced goodness-of-fit values indicative of poor model fit (χ^2^ > 10) and suggested CaCO_3_ abundances vary between otoliths in a pair. In this light, neutron diffraction data were also refined with independent refinement parameters (i.e., refinement paraments for otoliths in a pair were independent). Independent refinement parameters frequently produced better goodness-of-fit values, indicating polymorph abundances are variable within otolith pairs. Diffraction patterns for each otolith were refined using least squares regression, and *Χ*^2^ values less than ten were deemed a ‘good’ fit.

### Polymorph phase fraction data analysis

Paired t-tests were used to evaluate the differences in polymorph abundances quantified using neutron diffraction between each pair of otoliths. We then fit a least-squares regression line between the percent composition of otoliths ‘A’ and ‘B’ and tested for differences between the slope of experimental data and the slope of a 1:1 line (i.e., the line that would be expected if A and B otoliths were identical in polymorph composition). We did not attempt to make associations between fish characteristics (i.e., time spent in ocean/ freshwater, length, sex) and percent polymorph composition due to the limited sample size. Tests were considered significant at α = 0.05 level.

### Neutron diffraction validation with micro-Raman spectroscopy

Micro-Raman spectroscopy was used to help validate the presence of polymorphs indicated by neutron diffraction. Unlike neutron diffraction, Raman spectroscopy can provide information on the spatial distribution of CaCO_3_ polymorphs; however, it only provides information about surficial mineralogy (while laser penetration depth is variable, peak position(s) and intensity will primarily reflect the upper most portion of an otolith, ca. 1–3 µm depth) and cannot readily quantify mineral abundances. Raman scans were conducted with a 532 nm laser operating at 50% power. The topography of bulk otoliths required a 50 × objective to ensure the assessed areas were in focus. Importantly, scan locations were selected based on sample topography, not inferred mineralogy, that is, we conducted Raman spectroscopy on otolith areas that fit the general descriptions in the literature of different polymorphs (e.g., vaterite is translucent, calcite is chalky). These regions were analyzed by sampling one point per 10 μm × 10 μm area across a user-defined grid or manual point selection. In either case, five Raman spectra were acquired at each location.

Raman spectra frequently exhibit broadened, split, or shifted peaks that may reflect the presence of two or more polymorphs within the assessed region. Nondescript or broadened Raman peaks may result from the presence of multiple polymorphs within the interaction volume of the beam (i.e., within the diameter or penetration depth of the laser). Individual CaCO_3_ polymorphs were differentiated by the identification of two or more prominent peaks that are diagnostic of a specific CaCO_3_ polymorph (Fig. 6). If two or more sets of diagnostics peaks were identified at an assessment site, the peak set with greater intensities was recorded; if diagnostic peak sets could not be identified or confidently differentiated from other peak sets, data for that location were discarded. The cyanoacrylate glue and neutron absorbing coating used to adhere otoliths to the motorized gimble during neutron diffraction analysis were also assessed with micro-Raman to ensure residual amounts of these compounds did not interfere with peak identification. Neither material created Raman peaks resembling those of CaCO_3_ polymorphs.

### Phase confirmation with polarized light microscopy

Standard polished Sects. (30 um thick) were made for selected otoliths to show the microstructural and textural relations of the aragonite, vaterite and calcite (e.g., Fig. 4 bottom, sample 5244-b). If sufficient in grain size, aragonite, calcite and vaterite each have distinctive optical properties that allow them to be distinguished in polished thin sections using polarized light microscopy. The large crystal size of the calcite allows optical interference figures in conoscopic viewing and the characteristic uniaxial (−) optic sign is confirmed. The grain size was too small to confirm the optical properties of the vaterite (uniaxial +) and aragonite (biaxial −).

## References

[1] Morrongiello JR, Thresher RE, Smith DC (2012). Aquatic biochronologies and climate change. Nat. Clim. Change.

[2] Pracheil BM, Hogan JD, Lyons J, McIntyre PB (2014). Using hard-part microchemistry to advance conservation and management of North American freshwater fishes. Fisheries.

[3] Starrs D, Ebner BC, Fulton CJ (2016). All in the ears: Unlocking the early life history biology and spatial ecology of fishes. Biol. Rev..

[4] Limburg KE (1995). Otolith strontium traces environmental history of subyearling American shad *Alosa sapidissima*. Mar. Ecol. Progr. Ser..

[5] Kennedy BP, Klaue A, Blum JD, Folt CL, Nislow KH (2002). Reconstructing the lives of fish using Sr isotopes in otoliths. Can. J. Fish. Aquat. Sci..

[6] Hogan JD, Blum MJ, Gilliam JF, Bickford N, McIntyre PB (2014). Consequences of alternative dispersal strategies in a putatively amphidromous fish. Ecology.

[7] Carlson AK, Phelps QE, Graeb BDS (2017). Chemistry to conservation: using otoliths to advance recreational and commercial fisheries management. J. Fish Biol..

[8] Campana SE (1999). Chemistry and composition of fish otoliths: pathways, mechanisms and applications. Mar. Ecol. Prog. Ser..

[9] Pracheil BM, Chakoumakos BC, Feygenson M, Whitledge GW, Koenigs RP, Bruch RM (2017). Sturgeon and paddlefish (Acipenseridae) sagittal otoliths are composed of the calcium carbonate polymorphs vaterite and calcite. J. Fish Biol..

[10] Pracheil BM, George R, Chakoumakos BC (2019). Significance of otolith calcium carbonate crystal structure diversity to microchemistry studies. Rev. Fish Biol. Fish..

[11] Nehrke G, Poigner H, Wilhelms-Dick D, Brey T, Abele D (2012). Coexistence of three calcium carbonate polymorphs in the shell of the Antarctic clam Laternula elliptica. Geochem. Geophys. Geosyst..

[12] Wassenburg JA, Scholz D, Jochum KP, Cheng H, Oster J, Immenhauser A, Richter DK, Haeger T, Jamieson RA, Baldini JUL, Hoffmann D (2016). Determination of aragonite trace element distribution coefficients from speleothem calcite–aragonite transitions. Geochim. Cosmochim. Acta.

[13] Tzeng WN, Chang CW, Wang CH, Shiao JC, Iizuka Y, Yang YJ, You C-F, Ložys L (2007). Misidentification of the migratory history of anguillid eels by Sr/Ca ratios of vaterite otoliths. Mar. Ecol. Prog. Ser..

[14] Gauldie RW (1996). Effects of temperature and vaterite replacement on the chemistry of metal ions in the otoliths of *Oncorhynchus tshawytscha*. Can. J. Fish. Aquat. Sci..

[15] Reimer T, Dempster T, Wargelius A, Fjelldal PG, Hansen T, Glover KA, Solberg MF, Swearer SE (2017). Rapid growth causes abnormal vaterite formation in farmed fish otoliths. J. Exp. Biol..

[16] Coll-Lladó C, Giebichenstein J, Webb PB, Bridges CR (2018). Ocean acidification promotes otolith growth and calcite deposition in gilthead sea bream (*Sparus aurata*) larvae. Sci. Rep..

[17] Loeppky AR, Belding LD, Quijada-Rodriguez AR, Morgan JD, Pracheil BM, Chakoumakos BC, Anderson WG (2021). Influence of ontogenetic development, temperature, and pCO2 on otolith calcium carbonate polymorph composition in sturgeons. Sci. Rep..

[18] Melancon S, Fryer BJ, Ludsin SA, Gagnon JE, Yang Z (2005). Effects of crystal structure on the uptake of metals by lake trout (*Salvelinus namaycush*) otoliths. Can. J. Fish. Aquat. Sci..

[19] Veinott GI, Porter TR, Nasdala L (2009). Using Mg as a proxy for crystal structure and Sr as an indicator of marine growth in vaterite and aragonite otoliths of aquaculture rainbow trout. Trans. Am. Fish. Soc..

[20] Loeppky AR, Chakoumakos BC, Pracheil BM, Anderson WG (2019). Otoliths of sub-adult Lake Sturgeon Acipenser fulvescens contain aragonite and vaterite calcium carbonate polymorphs. J. Fish Biol..

[21] Vignon M (2020). When the presence of a vateritic otolith has morphological effect on its aragonitic partner: Trans-lateral compensation induces bias in microecological patterns in one-side-only vateritic otolith. Can. J. Fish. Aquat. Sci..

[22] Clarke AD, Telmer KH, Mark Shrimpton J (2007). Elemental analysis of otoliths, fin rays and scales: A comparison of bony structures to provide population and life-history information for the Arctic grayling (*Thymallus arcticus*). Ecol. Freshw. Fish.

[23] Campana SE, Chouinard GA, Hanson JM, Frechet A, Brattey J (2000). Otolith elemental fingerprints as biological tracers of fish stocks. Fish. Res..

[24] Gauldie RW (1993). Continuous and discontinuous growth in the otolith of Macruronus novaezelandiae (Merlucciidae: Teleostei). J. Morphol..

[25] Long JM, Snow RA, Pracheil BM, Chakoumakos BC (2021). Morphology and composition of Goldeye (Hiodontidae; Hiodon alosoides) otoliths. J. Morphol..

[26] Chakoumakos BC, Pracheil BM, Koenigs RP, Bruch RM, Feygenson M (2016). Empirically testing vaterite structural models using neutron diffraction and thermal analysis. Sci. Rep..

[27] David AW, Grimes CB, Isely JJ (1994). Vaterite sagittal otoliths in hatchery-reared juvenile red drums. Progres. Fish-Cult..

[28] Tomás J, Geffen AJ (2003). Morphometry and composition of aragonite and vaterite otoliths of deformed laboratory reared juvenile herring from two populations. J. Fish Biol..

[29] Kamhi SR (1963). On the structure of vaterite CaCO3. Acta Crystallogr. A.

[30] Kartnaller V, Ribeiro EM, Venancio F, Rosariob F, Cajaiba J (2018). Preferential incorporation of sulfate into calcite polymorphs during calcium carbonate precipitation: an experimental approach. CrystEngComm.

[31] Paquette J, Reeder RJ (1995). Relationship between surface structure, growth mechanism, and trace element incorporation in calcite. Geochim. Cosmochim. Acta.

[32] Hüssy K, Mosegaard H (2004). Atlantic cod (Gadus morhua) growth and otolith accretion characteristics modelled in a bioenergetics context. Can. J. Fish. Aquat. Sci..

[33] Fablet R, Pecquerie L, De Pontual H, Høie H, Millner R, Mosegaard H, Kooijman SA (2011). Shedding light on fish otolith biomineralization using a bioenergetic approach. PLoS ONE.

[34] Naslund AW, Davis BE, Hobbs JA, Fangue NA, Todgham AE (2021). Warming, not CO2-acidified seawater, alters otolith development of juvenile Antarctic emerald rockcod (*Trematomus bernacchii*). Polar Biol..

[35] Coll-Lladó C, Mittermayer F, Webb PB, Allison N, Clemmesen C, Stiasny M, Göttler G (2021). Pilot study to investigate the effect of long-term exposure to high pCO2 on adult cod (*Gadus morhua*) otolith morphology and calcium carbonate deposition. Fish Physiol. Biochem..

[36] Söllner C, Burghammer M, Busch-Nentwich E, Berger J, Schwarz H, Riekel C, Nicolson T (2003). Control of crystal size and lattice formation by starmaker in otolith biomineralization. Science.

[37] Rodriguez-Carvajal, J. FULLPROF: A program for Rietveld refinement and pattern matching analysis. In *Satellite Meeting on Powder Diffraction of the XV Congress of the IUCr* (Vol. 127) (1990).

[38] Roisnel T, Rodríquez-Carvajal J (2001). WinPLOTR: A windows tool for powder diffraction pattern analysis. Mater. Sci..

[39] Momma K, Izumi F (2008). VESTA: A three-dimensional visualization system for electronic and structural analysis. J. Appl. Crystallogr..

[40] Slater JC (1964). Atomic radii in crystals. J. Chem. Phys..

